# The Structure of the LysR-type Transcriptional Regulator, CysB, Bound to the Inducer, *N*-acetylserine

**DOI:** 10.1007/s00249-024-01716-w

**Published:** 2024-07-08

**Authors:** Koen H. G. Verschueren, Eleanor J. Dodson, Anthony J. Wilkinson

**Affiliations:** 1 York Structural Biology Laboratory, Department of Chemistry, University of York, York, YO10 5DD UK; 2 Unit for Structural Biology, VIB Center for Inflammation Research, Ghent, Belgium; Unit for Structural Biology, Department of Biochemistry and Microbiology, Ghent University, Ghent, Belgium

**Keywords:** CysB, Cysteine biosynthesis, LysR-family, Crystal structure, Transcriptional regulator

## Abstract

In *Escherichia coli* and *Salmonella typhimurium*, cysteine biosynthesis requires the products of 20 or more *cys* genes co-ordinately regulated by CysB. Under conditions of sulphur limitation and in the presence of the inducer, *N*-acetylserine, CysB binds to *cys* promoters and activates the transcription of the downstream coding sequences. CysB is a homotetramer, comprising an N-terminal DNA binding domain (DBD) and a C-terminal effector binding domain (EBD). The crystal structure of a dimeric EBD fragment of CysB from *Klebsiella aerogenes* revealed a protein fold similar to that seen in Lac repressor but with a different symmetry in the dimer so that the mode of DNA binding was not apparent. To elucidate the subunit arrangement in the tetramer, we determined the crystal structure of intact CysB in complex with *N*-acetylserine. The tetramer has two subunit types that differ in the juxtaposition of their winged helix-turn-helix DNA binding domains with respect to the effector binding domain. In the assembly, the four EBDs form a core with the DNA binding domains arranged in pairs on the surface. *N*-acetylserine makes extensive polar interactions in an enclosed binding site, and its binding is accompanied by substantial conformational rearrangements of surrounding residues that are propagated to the protein surface where they appear to alter the arrangement of the DNA binding domains. The results are (i) discussed in relation to the extensive mutational data available for CysB and (ii) used to propose a structural mechanism of *N*-acetylserine induced CysB activation.

**Supplementary Information:**

The online version contains supplementary material available at 10.1007/s00249-024-01716-w.

## Introduction

Cysteine biosynthesis in Gram-negative bacteria involves the products of more than 20 *cys* genes arranged in operons distributed around the chromosome. These *cys* genes are associated with the uptake of sulphate from the environment and its reduction to sulphide. The *cys* regulon also includes the genes encoding serine acetyl transferase, which catalyses the activation of serine by O-acetylation, and the gene encoding *O*-acetylserine thiol lyase which catalyses the reaction of sulphide with *O*-acetylserine producing L-cysteine and acetate (Kredich 2008; Verschueren and Wilkinson 2005). Thiosulphate is an alternative source of sulphur in cysteine biosynthesis. It reacts with *O*-acetylserine to form *S*-sulphocysteine and acetate with the former converted to cysteine and sulphite by glutaredoxin-1 and the glutaredoxin-like protein NrdH (Nakatani et al. 2012). These reactions represent the primary routes through which these bacteria fix sulphur for use in numerous metabolic processes which depend on this element. CysB also regulates a CysB-like protein Cbl that is required for utilisation of alkanesulfonates (Iwanicka-Nowicka and Hryniewicz 1995; Stec et al. 2006).

Cysteine biosynthesis is regulated in a complex way (Kredich 1992). Cysteine, itself, is an inhibitor of serine acetyltransferase, which leads to a decrease in the concentration of the precursor, *O*-acetylserine. At the transcriptional level, many of the *cys* genes are co-ordinately regulated by the activator protein, CysB. CysB is a homotetramer of 36 kDa subunits, each comprising a smaller N-terminal DNA binding domain and a C-terminal effector binding domain (Miller and Kredich 1987; Hryniewicz and Kredich 1994). CysB binds at sequences upstream of the -35 region of its target promoters and in the presence of inducer activates transcription (Kredich 1992). DNase I footprinting experiments showed that CysB protects extended regions of the DNA and data on the stoichiometry of binding led to the proposal of schemes based on multiple 19 base pair binding half-sites (Hryniewicz and Kredich 1994, 1995; Monroe et al. 1990). These sites vary in their number, orientation, and position with respect to the transcription start site at different promoters. These binding site arrangements correlate with different effects of cofactor binding which can involve increasing or decreasing the affinity of CysB for DNA or altering the extent of CysB-induced DNA bending at *cys* promoters. Sulphide and thiosulphate act as anti-inducers which prevent activation by *N*-acetylserine when sulphur is not limiting (Hryniewicz and Kredich 1991; Ostrowski and Kredich 1990). At the *cysB* promoter, CysB binds to a site overlapping the RNA polymerase binding site leading to transcriptional repression. In the presence of *N*-acetylserine, which is formed spontaneously from *O*-acetylserine, CysB binding to this promoter is reduced and repression is relieved (Ostrowski and Kredich 1991).

CysB is a member of the LysR-type transcriptional regulator (LTTR) family, named after its founding member (Stragier et al. 1983; Henikoff et al. 1988). The LTTRs exhibit sequence similarities distributed over 300 or so residues which is most pronounced in the N-terminal ~ 65 residues which constitute a DNA binding domain. The LTTRs are widely distributed and represent one of the largest families of transcriptional regulators in bacteria, with some 45 members identified in *Escherichia coli* (Perez-Rueda and Collado-Vides 2000; Pareja et al. 2006). They regulate diverse aspects of cellular metabolism, function and responses to environmental change (Schell 1993; Baugh et al. 2023). Like CysB, most family members activate transcription at one or more *loci* whilst negatively regulating the expression of their own gene. Though not the case for CysB, the target genes of many LTTRs are closely linked to, and divergently transcribed from, the gene encoding the LTTR itself.

The crystal structure of a dimeric cofactor binding domain fragment of CysB from *Klebsiella aerogenes* (KaCysB) (residues 88–324) gave the first detailed insights into tertiary and quaternary structure in the LysR family proteins (Tyrrell et al. 1997). It revealed a cofactor-binding site containing a sulphate ion enclosed by two lobes in a manner reminiscent of the solute binding proteins of ABC transporters and transcription factors belonging to the Lac repressor family (Tyrrell et al. 1997; Verschueren and Wilkinson 2003) (Supplementary Fig. 1A). However, the subunit organisation in the dimer was different to that in Lac repressor (Friedman et al. 1995) (Supplementary Fig. 1B). Instead of the two-fold symmetry axis relating the subunits in the dimer aligning with their long axis, it was orthogonal to this direction, such that the N-terminal DNA binding domains would be attached at opposite ends of the dimer. It was not clear how the subunits would be arranged in the tetramer, nor how the four DNA-binding domains would be juxtaposed to account for the complex pattern of DNase I footprints produced by CysB at its various target promoters in the presence and absence of inducer.

To understand the organisation of the subunits in the intact protein, we grew crystals of KaCysB in the presence of *N*-acetylserine and collected diffraction data from different crystal forms (Verschueren et al. 2001). Despite intensive efforts, we were unable to solve their structure. In the meantime, other structures of full-length LTTRs were determined, the first being CbnR, the regulator of chlorocatechol degradation in *Ralstronia eutropha* (Muraoka et al. 2003a) quickly followed by DntR, a regulator of nitrotoluene degradation in Burkhoderia sp. (Smirnova et al. 2004) and later many more (Baugh et al. 2023; Momany and Neidle 2012). Here, we have revisited the CysB crystallographic data using improved molecular replacement methods and solved the structure of the CysB tetramer relatively straightforwardly. The structure and the mode of *N*-acetylserine binding are presented and discussed in relation to (i) the abundant genetic and biochemical data accumulated for the CysB orthologues in *Salmonella typhimurium* and *E. coli* and (ii) the structural basis of *N*-acetylserine induction of the cysteine regulon.

## Methods

### Structure Solution

Full-length CysB(1–324) was crystallised in the presence of *N*-acetylserine in two different crystal forms that were visually indistinguishable and crystallographically related (Verschueren et al. 2001). The *R*32 (now *H*32) crystals diffracted to a resolution of 2.3 Å and were predicted to have two molecules in the asymmetric unit. For the *R*3 (now *H*3) form, which diffracted to 2.8 Å spacing, there is a doubling of the *c* axis which requires that the space group is reindexed as *H*3 with the H3 *a* axis overlapping the *H*32 *a* + *b*. This breaks the *H*32 two fold symmetry so the number of molecules per asymmetric unit is quadrupled. However, the molecular interactions remain exactly the same (Table 1).

**Table 1:** Data collection and refinement statistics

PDB ID	9F14	9FDD
Crystallization conditions	12–17% PEG8000_,_ 0.2 M sodium tartrate_,_ 0.1 M Tris–HCl pH 8.5, 0.2 M KSCN_,_ 10 mM NAS	12–17% PEG8000_,_ 0.2 M sodium tartrate_,_ 0.1 M Tris–HCl pH 8.5_,_ 10 mM NAS
Protein Conc^n^	4–5 mg / mL	4–5 mg / mL
Data collection		
X-ray source (beamline)	SRS Daresbury (9.6)	SRS Daresbury (9.6)
Wavelength (Å)	0.87	0.87
Space group	*H32*	*H3*
Cell dimensions		
* a*, *b*, *c* (Å)	185.86, 185.86, 113.04	187.44, 187.44, 224.66
α,β,γ (°)	90.00, 90.00, 120.00	90.00, 90.00, 120.00
Resolution (Å)^a^	19.81—2.30 (2.38—2.30)	129.10–2.80 (2.86–2.80)
Unique reflections^a^	33 205 (3 251)	72 368 (4 472)
* R*_meas_^a,b^	7.2 (57.2)	8.9 (35.6)
* I*/σ*I* ^a^	20.0 (3.4)	18.8 (3.8)
CC ½ (%) ^a^	99.9 (96.0)	n.a
Completeness (%) ^a^	99.8 (100.0)	99.9 (100.0)
Redundancy^a^	11.6 (11.6)	3.8 (1.5)
Wilson B factor	43.18	57.20
Refinement		
Resolution (Å)	19.81—2.30	41.43 – 2.80
No. reflections	33 193	72 368
* R*_work_ / *R*_free_^c,d^	19.71 / 24.59	20.00 / 22.99
No. atoms		
Protein	5 072	20 439
Ligand/ion	20	80
Water	242	44
* B*-factors		
Protein	64.8	48.00
Ligand/ion	57.8	31.50
Water	52.7	26.05
R.m.s. deviations^e^		
Bond lengths (Å)	0.008	0.008
Bond angles (°)	0.91	0.95

^a^ Values in parentheses correspond to the outer resolution shell

^b^ *R*_meas_ = ∑_*hkl*_(n/(n-1))^1/2^∑_*i*_|*I*_*i*_*—*< *I* >|*/*∑_*hkl*_∑_*i*_ < *I* > where *I*_*i*_ is the intensity of the *i*th measurement of a reflection with indexes *hkl* and < *I* > is the statistically weighted average reflection intensity

^c^ *R*_work_ = ∑||*F*_*o*_|*—*|*F*_*o*_||/∑|*F*_*o*_| where *F*_*o*_ and *F*_*c*_ are the observed and calculated structure factor amplitudes, respectively

^d^ *R*_free_ is the *R*-factor calculated with 5% of the reflections chosen at random and omitted from refinement

^e^ Root-mean-square deviation of bond lengths and bond angles from ideal geometry

The structure of CysB(1–324) in space group *H*32 was elucidated by molecular replacement using MOLREP (Vagin and Teplyakov 2010) with the ligand-binding domain, residues 88–324 (PDB: 1AL3), as the search model. The two copies of this chain found in the asymmetric unit form a dimer related by two-fold non-crystallographic symmetry. The arrangement of this dimer corresponds to the crystallographic dimer observed for CysB(88–324). The resulting phases were good enough to allow the ‘missing’ DNA-binding domains (residues 1–87) to be built by BUCCANEER (Cowtan 2006) implemented in the CCP4i2 package (Potterton et al. 2018). Model (re)building was performed in Coot and refinement of coordinates and atomic displacement parameters was performed in autoBUSTER (http://www.globalphasing.com/buster/) and REFMAC (Murshudov et al. 1997). Model and map validation tools in Coot, the CCP4 package and the PDB_REDO server were used throughout the work flow to guide improvement and to validate the quality of crystallographic models. For the two molecules, A and B, in the asymmetric unit of this crystal form, the DNA-binding domains have a different conformation relative to the ligand binding domains which allows them to form an interface with a pair of symmetry related molecules. A tetramer can be generated from chains A and B by applying the symmetry operator 2/3-X, 1/3-X + Y, 1/3-Z. This tetramer was then used in molecular replacement searches of the *H*3 space group, placing two tetramers, chains ABCD and EFGH, in the *H*3 asymmetric unit. The tetramer structures in the two crystal forms are very similar, with root mean square difference (rmsΔ) values of 0.62 Å for 1296 equivalent residues in the *H*32 and *H*3 forms.

## Results & Discussion

### Overall Structure

**Fig. 1:**
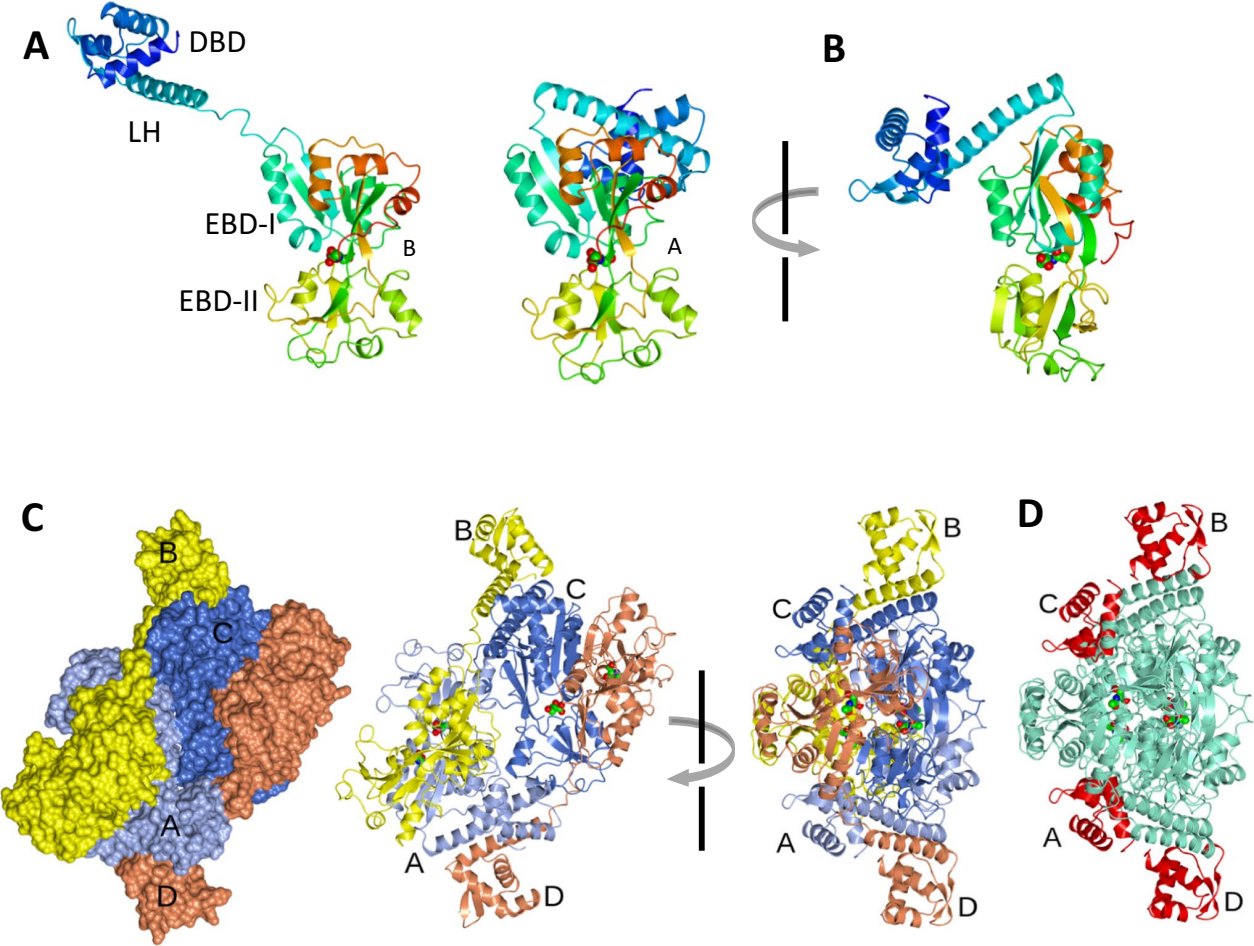
Tertiary and quaternary structure of CysB. **A** Extended (chain B) and compact (chain A) CysB protomers. The chains are colour ramped from their N-termini (blue) to their C-termini (red). The orientations of the effector binding domains are similar emphasising the alternate juxtapositions of the linker and DNA binding domains. The *N*-acetylserine ligands are shown in sphere representation coloured by atom type. **B** An orthogonal view of chain A in which the DNA binding domain is not eclipsed is shown. **C** Surface (left) and ribbon (centre) representations of the ABCD CysB tetramer from the *H*3 crystal structure. The colouring is by chain A, ice blue; chain B, yellow; chain C, light blue; chain D, coral. An orthogonal view of the CysB tetramer in ribbon representation is shown on the right. **D** The CysB tetramer oriented as in the right panel of **C** and with the DNA binding domains coloured in red and the ligand binding domains coloured in aquamarine

The *H*32 and *H*3 crystal structures have 2 and 8 molecules in the asymmetric unit respectively. In the *H*32 crystal form, a crystallographic twofold axis of symmetry generates the CysB tetramer while the asymmetric unit of the *H*3 crystal form contains two CysB tetramers. The 324 amino acid residue CysB protomers form a structure comprising three key elements (Fig. 1A). An N-terminal DNA binding domain (DBD) leads into a linker α-helix (LH) that connects to the effector binding domain (EBD) which is composed of two subdomains which we will refer to as EBD-I and EBD-II to be consistent with a recent authoritative review of structure and activity of the LTTR family proteins (Baugh et al. 2023). There are two protomer types in each tetramer distinguished by the juxtaposition of the DNA binding and the effector binding domains (Fig. 1A & B). In each tetramer (Fig. 1C), two of the chains (B and D) adopt the extended conformation as illustrated for Molecule B in Fig. 1A while the other two chains (A and C) exhibit a compact conformation as shown for Molecule A in Fig. 1B. In both chain types, the DNA binding domains are closely superimposable as are the effector binding domains. The change in overall structure arises from alternative conformations of hinge regions flanking the linker helix (Fig. 1A & B). In the tetramer as shown in Fig. 1D, the four effector binding domains form a core from which the DNA binding domains project in pairwise arrangements, A-D and B-C.

### The structure of the DNA binding domain

**Fig. 2:**
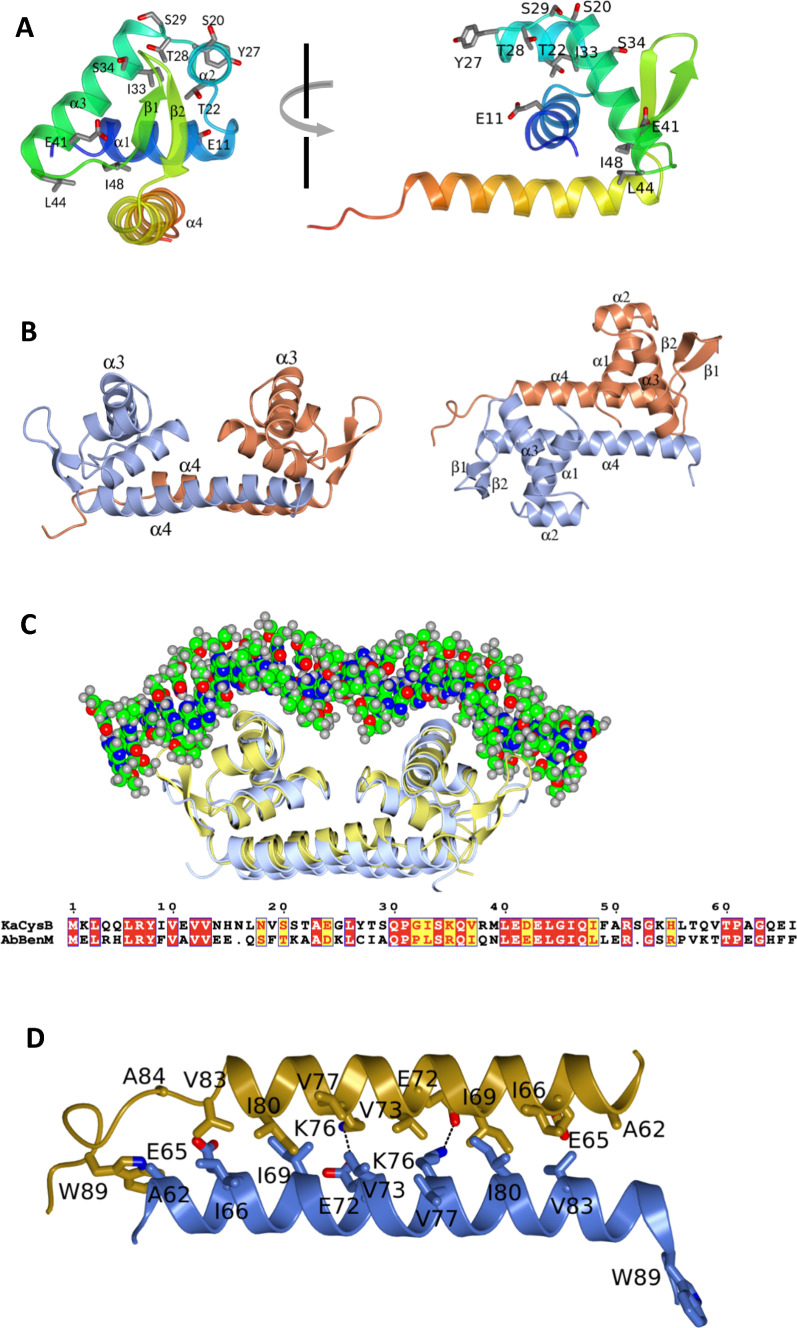
DNA Binding Domain. **A** Orthogonal views of the DNA binding domain of chain D. The chain is in ribbon representation and colour ramped from residue 1 (blue) to residue 88 (red). Secondary structure elements are labelled in the left panel and side chains of residues whose mutation affects CysB function are shown with labels in both panels. **B** The linker regions and DNA binding domains of chains A (ice blue) and D (coral) shown in approximately orthogonal views. **C** Superposition of the AD LH-DBD pair from CysB (gold) with the AB linker-DBD domain pair (ice blue) and associated DNA (spheres coloured by element) from the crystal structure of the BenM-DNA complex (4IHT) (Giannopoulou et al. 2021). Resides 1–66 of the respective protein chains, aligned in the lower panel, were overlaid using the Superpose Models routine in the programme CCP4mg producing an rmsΔ value of 1.3 Å for 122 equivalent C_α_ atoms. **D** Detail of the packing of the linker helices in the AD interface with residues labelled and ionic interactions indicated by dashed lines

The N-terminal 87 residues form a winged helix-turn-helix (wHTH) domain structure consistent with (i) their function in DNA binding and (ii) the structures determined of DNA binding domains (DBDs) of other LTTRs (Muraoka et al. 2003b; Alanazi et al. 2013). The domain comprises four α–helical segments and a β–hairpin in the order α1–α2–α3–β1–β2–α4 with α4 also constituting the linker helix (Fig. 2A). In common with many sequence specific DNA binding proteins, the DBDs are arranged in pairs with local two-fold symmetry (Fig. 2B). Helices α2 and α3, spanning residues 19–44, constitute the scaffolding and recognition elements of the helix-turn-helix motif respectively with the β1–β2 element constituting the wing. These motifs are expected to mediate sequence specific DNA recognition. An insight into how this might take place can be gained from superposing the DNA-binding domain in the CysB AD dimer onto the crystal structure of the DBD of BenM bound to DNA (Alanazi et al. 2013). As illustrated in Fig. 2C, the recognition helix binds in the major groove of the DNA with the wing element binding in the adjacent minor groove.

Besides its contribution to the structure of the wHTH domain, helix α4 is an important determinant of the quaternary structure of CysB. The α4-helices of chains A and D (as well as those of chains B and C) align in an anti-parallel sense, their association stabilised by coiled-coil interactions described in more detail below (Fig. 2D). The local two-fold symmetry of the DNA binding regions breaks down as α4 extends to Trp89 in chain A but gives way to random coil at Val83 in chain D with important implications for the structure of the CysB tetramer.

### Interfaces between the subunits

In the homotetramer (Fig. 1), there are six potential subunit interfaces. The molecular packing was analysed in the program PISA (Krissinel and Henrick 2007). Subunits B and D, which are both in the extended conformation, have no shared interface.

**Fig. 3:**
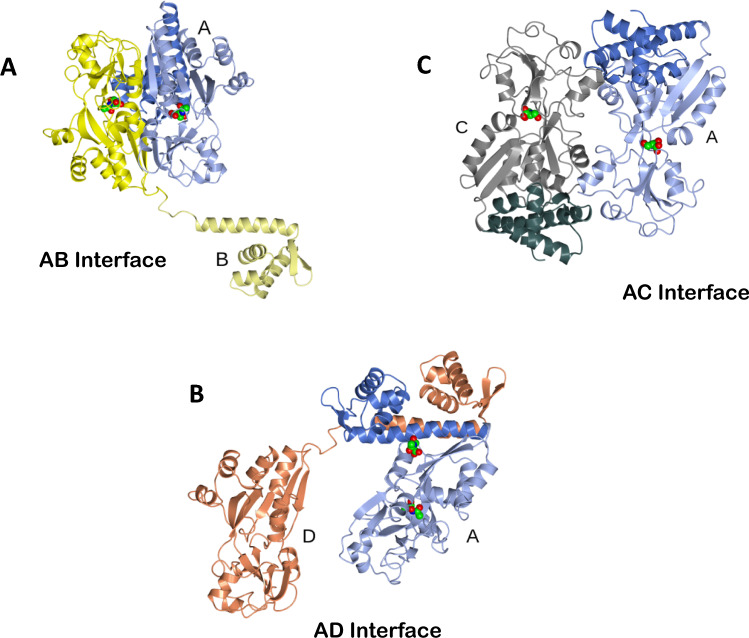
Interfaces between the subunits in the CysB tetramer. The colouring is by chain: A, blue; B, yellow; C, grey; and D coral. In each case, the DNA binding domain is emphasised by altered shading. **A** The AB interface which is dominated by EBD-EBD interactions is augmented by contacts made by the EBD of chain B and the linker and DBD regions of chain A. **B** The AD interface which is dominated by interactions between the linker helices supplemented by interactions of these helices with the partner DNA binding domains. **C** The AC interface viewed down a twofold symmetry axis features a prominent EBD-EBD interface with additional contacts between the EBDs and the linker helices

The interface between subunits A and B is extensive at 1540 Å^2^ with 45 residues on each chain participating (Fig. 3A). The regulatory domains of these two subunits are related by a local non-crystallographic two-fold axis of symmetry which is perpendicular to the long axis of the effector binding domains. Thus, the first regulatory subdomain (EBDI) of chain A comes together with the second regulatory subdomain (EBDII) of chain B with main chain hydrogen bonding between strand β_B_ of chain A and β_G_ of chain B which extends the five-stranded β-sheets in the respective domains to form a 10-stranded intermolecular β-sheet. These contacts are duplicated through the interactions of EBDI of chain B with EBDII of chain A. This interface is augmented by interactions of residues from the EBDII domains of chains A and B respectively with residues 88–90 in the linker region of chain B and residues (Asn16, Glu24 and Tyr27) in the DNA binding domain of chain A. The interactions between chain A and B are closely similar to those between the quasi-equivalent pair of chains D and C respectively. This interface corresponds closely to that between the two chains of the dimer in the crystal structure of CysB(88–324) (PDB: 1AL3).

The interactions between chains A and D, equivalent to those between chains C and B, are dominated by the packing of the linker helices. These helices which align in an anti-parallel sense, are stabilised by hydrophobic interactions at heptadic repeat positions. Ala62, Glu65, Ile66, Ile69, Glu72, Val73, Lys76, Ile80 and Val83 from each subunit contribute significantly to the apolar surface area buried in the interface with additional stabilisation provided by reciprocal salt-bridges formed between Glu72 and Lys76 (Fig. 3B and 2D). Additional interactions are made between the α4 helices and the α3-β1 loops of the DNA binding domains of the partner subunit. Finally, the N-terminal residues Met1 and Leu3 are prominent in this interface. These interactions together bury 1160 Å^2^ of the surface area on each chain. The effector binding domains do not contribute significantly to this interface (Fig. 3B).

The interface between subunits A and C, which are both in the compact conformation (Fig. 3C), is more modest with an area of 775 Å^2^. Residues Gln64, Glu65 and Arg68 at the N-terminus of the linker helix (α4) of subunit A pack together with residues Gly215, Leu216, Thr217 and Arg219 on the partner subunit C, with Glu65 and Arg219 forming an ion pair. Meanwhile residues Asp156 and 157 together with Arg307, Ser308 and Glu310 form a surface on EBDI of subunit A that packs against a surface of the EBDII of subunit C formed by Thr209, Asn212 and Arg213, with Glu310 and Arg213 forming and ion pair. As a result of the two-fold symmetry, reciprocal interactions are made between subunit C and subunit A. Finally, the side chains of residues Arg204, which are situated close to the symmetry axis at the heart of the tetramer, pack against one another. This interface is extended but distributed into multiple patches. In their totality, the interactions between subunits A and C are weak with a low complex formation significance score of 0.033 according to PISA (Krissinel and Henrick 2007).

Analysis of the subunit interactions provides a clear explanation for the dimeric structure of CysB(88–324) which was produced by limited chymotryptic digestion of the full length protein. The resulting dimer fragments are derived from the AB (or CD) subunits. The interface between these regulatory domain pairs is extensive and sustains these subunit interactions. In the tetramer, the interface between the A and C effector binding domains is smaller and insufficient to sustain the tetramer organisation upon chymotryptic proteolysis around residue 88 and loss of the linker helices and the DNA binding domains which sustain the interactions of subunits A and D and B and C.

### N-acetylserine binding

**Fig. 4:**
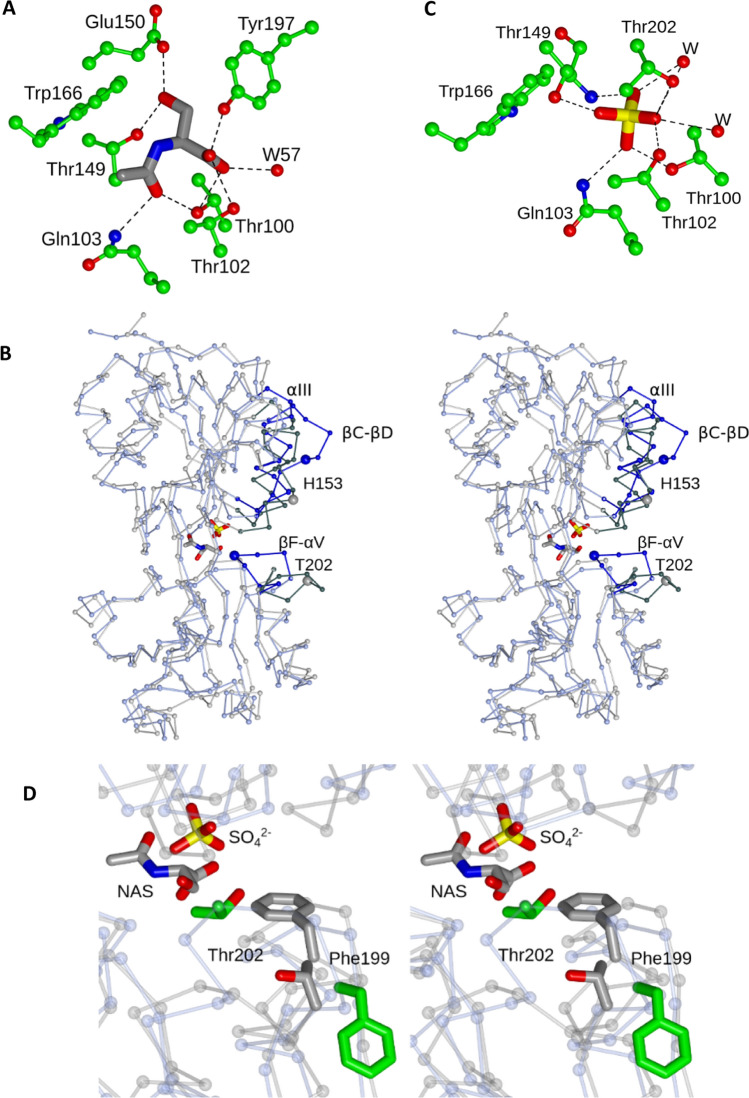
*N*-acetylserine Binding. **A** The *N*-acetylserine ligand in the A molecule of the *H*32 crystal form is shown in cylinder format with neighbouring protein side chains and a water molecule (W) in ball-and-stick format. Atoms are coloured by element with the carbons of *N*-acetylserine in grey and the protein carbons in green. Polar interactions between the protein and ligand are indicated by dashed lines. **B** Stereo C_α_ superposition of residues 90–324 of CysB(88–324)-sulphate (ice blue) on to the equivalent residues of the A chain from the *H3*2 structure of CysB-NAS (grey). The C_α_s of residues His153 and Thr202 are enlarged and labelled. The regions of the structure which exhibit the largest differences are shown in darker shades and labelled. **C** Sulphate (cylinder format) bound in CysB(88–324) displayed together with surrounding residues (ball and stick) and with polar interactions indicated by dashed lines. **D** Stereo view of the ligand binding pockets of the overlaid CysB(88–324)-sulphate (ice blue C_α_s and green side chain carbon atoms) and CysB-NAS (grey C_α_s and grey side chain carbon atoms) structures with the side chains of Phe199 and Thr202 displayed to emphasise the structural changes these residues undergo when *N*-acetylserine binds

**Fig. 5:**
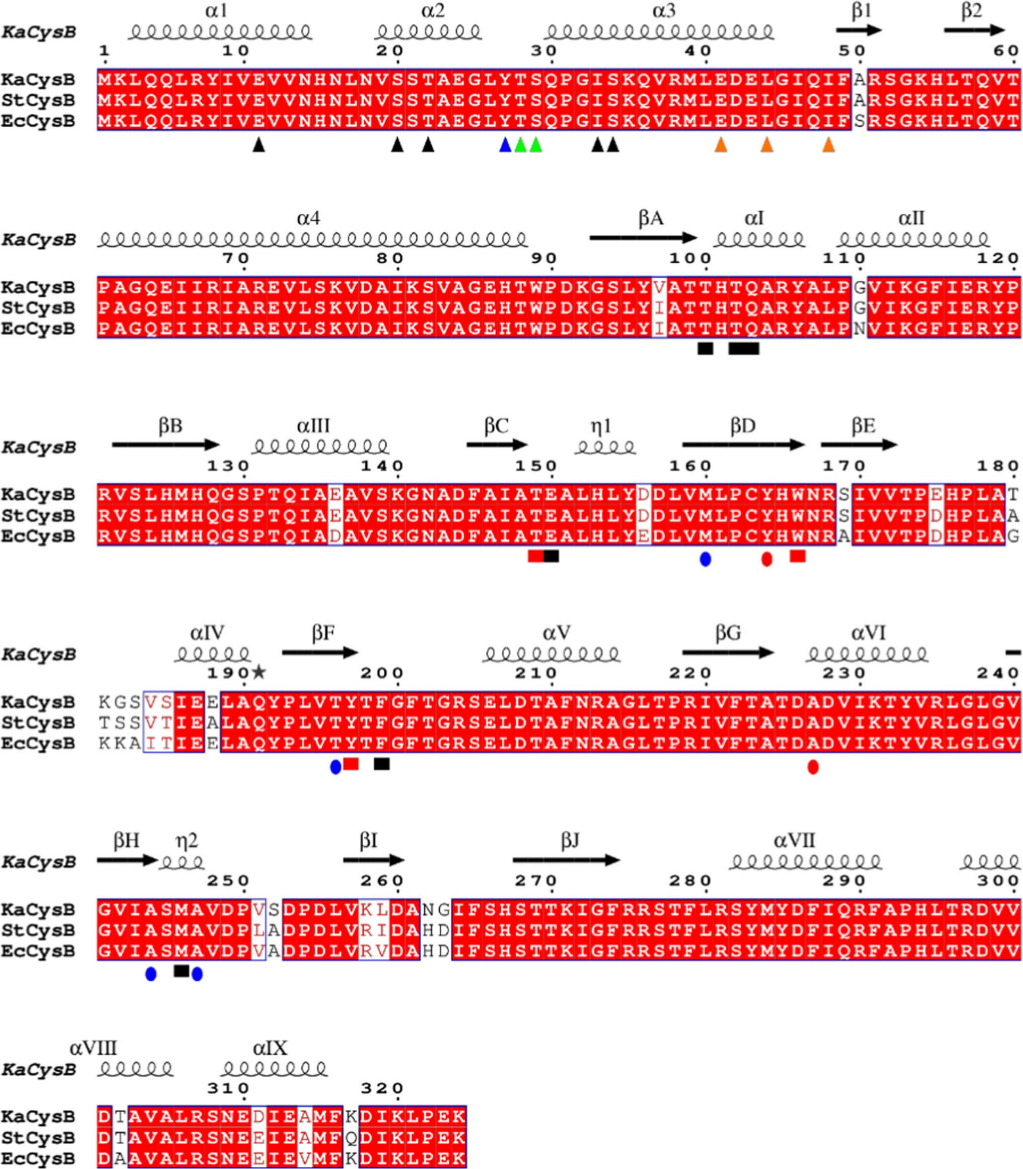
Alignment of CysB sequences. CysB sequences from *K. aerogenes* (Ka), *S. typhimurium* (St) and *E. coli* (Ec) with UNIPROT accession codes P45600, P06614 and P0A9F3 respectively were retrieved and aligned in ClustalW (Thompson et al. 1994) and displayed in the context of the KaCysB structure in the programme ESPRIPT (Robert and Gouet 2014). Sequence identities are indicated by the red filling. Shapes below the alignment indicate residues discussed in the text. Triangles denote sites of substitutions that affect DNA binding (black) transcriptional activation (green) or oligomerisation (orange). The blue triangle indicates a site where substitution can affect either DNA binding or *trans* activation. Squares indicate residues that form significant interactions with the ligand. These are coloured in red to denote sites of constitutive substitution mutations which are otherwise shown as red circles. Blue circles denote sites of non-inducible substitution mutations. The labelling of secondary structure elements in LTTRs was developed independently for the DBDs and EBDs. We have kept with this hybrid system as has been recommended (Baugh et al. 2023)

CysB(1–324) was crystallised in the presence of 10 mM of the inducer *N*-acetylserine (Verschueren et al. 2001). The ligand is enclosed in the prominent cleft between EBDI and EBDII (Fig. 1). It is generally well defined in the electron density maps of all of the subunits across the two crystal forms (Supplementary Fig. 2) though the occupancy is lower in the B molecule of the *H*32 crystal form. The NAS binding pocket is lined by residues Thr100, Thr102, Gln103, Thr149, Glu150, Trp166, Tyr197, Phe199 and Met246 as shown in Figs. 4A and 5 and Supplementary Fig. 2. The carbonyl oxygen of the *N*-acetyl group of the ligand accepts hydrogen bonds from the hydroxyl of Thr100 and the amide –NH_2_ of Gln103. The side chain of the serine forms a charge-dipole interaction with the Glu150 carboxylate and a dipole–dipole interaction with the hydroxyl of Thr149. Meanwhile, the carboxylate of the ligand forms interactions with the phenolic hydroxyl of Tyr197, the aliphatic hydroxyls of Thr100 and Thr102 and an ordered water molecule. Finally, the > N–H of the *N*-acetyl group is well placed to interact favourably with the π electron cloud of the indole ring of Trp166. The close proximity of Trp166 to the ligand in the crystal structure explains the large changes in protein fluorescence that accompany titration of KaCysB with *N*-acetylserine (Lynch et al. 1994). The close packing around the serine hydroxyl, and its solvation of the charge on Glu150 suggests the binding site would not readily accommodate the *N*-acetylserine precursor *O*-acetylserine, consistent with the earlier binding studies (Lynch et al. 1994; Fersht et al. 1985).

### Correlation of structure with mutagenesis data

Genetic studies have identified a rich pool of mutations in CysB which variously affect DNA binding, responses to cofactor, oligomerisation and transcriptional activation (Kredich 1992; Colyer and Kredich 1994; Lochowska et al. 2001). While these in vivo and in vitro functional studies have been carried out with the CysB orthologues from *E. coli* and *S. typhimurium*, they can be interpreted confidently in the framework of the structures presented here of CysB from *K. aerogenes* because the three proteins are closely related with 95% pairwise sequence identity as shown in Fig. 5.

In one study, a library of CysB encoding plasmids was mutagenised and screened for loss of β-galactosidase activity in a P*cysP*-*lacZ* fusion reporter strain (Lochowska et al. 2001). This led to the identification of point mutants that were unable to activate transcription from the *cysP* promoter. Ten of these, encoding the substitutions E11K, S20L, T22I, Y27G, E41K, L44R, I48T, M160I, T196I, A244V and A247E, were tested for their capacity to repress β-galactosidase production in a *cysB* promoter *lacZ* fusion reporter system. Six were unable to negatively autoregulate, pointing to DNA binding defects in the E11K, S20L, T22I, Y27G, E41K, L44R substitution mutants (Lochowska et al. 2001). These characteristics are shared with two previously identified mutants with the substitutions I33N, and S34R mutants (Colyer and Kredich 1996). The sites of these substitutions, which map to the DNA binding domain are displayed on the sequence and the structure of CysB in Fig. 5 and Fig. 2A respectively.

The failure of mutants with E41K, L44R, I48T substitutions to exert transdominant effects on transcription when co-expressed with wild type CysB led the authors to postulate that these mutations caused defects in protein oligomerisation (Lochowska et al. 2001). Analysis of the crystal structure shows that these residues are clustered in the α3–β1 turn that forms the interface between the DNA binding domains and the linker helices. Their side chains are partly, or completely, buried such that reversal of charge at positions 41 and 44 and the introduction of polarity at position 48 are likely to disrupt the stability of the DNA binding domain and the interface with the partner subunit (Lochowska et al. 2001).

In a later study, the same group proposed on the basis of data from alanine-scanning mutagenesis, that residues Y27, T28 and S29 comprise an activating region that mediates positive control of *cysP* transcription in the presence of acetylserine (Lochowska et al. 2004). Using a LexA based two-hybrid approach, they showed that together these residues contribute to a surface that interacts with the ‘273 determinant’ of the α-subunit of RNA polymerase. In the crystal structure, Y27, T28 and S29 are situated in the turn between helices α2 and α3 of the helix-turn-helix motif with the –OH of the serine forming an N-capping hydrogen bond on helix α3 (Fig. 2A). The three side chains are part of a contiguous surface consistent with a common role in gene activation.

Collectively, mutagenesis studies have implicated residues spanning the polypeptide segment 149–247 as mediating inducer responses. Thus, T149M/P, Y164N, W166R, Y197S, and A227D substitutions result in constitutive activity of CysB (Colyer and Kredich 1994; Lochowska et al. 2001). Each of the mutated residues, Thr149, Tyr164, Trp166, Tyr197 and Ala227, contributes to the surface of the NAS binding pocket in the crystal structure consistent with the mutagenesis observations. These ‘constitutive’ substitutions presumably drive the binding site conformation towards the activated state even in the absence of the inducer. Meanwhile, the mutations M160I, T196I, A244V and A247E were characterised as non-inducible (Lochowska et al. 2001). The Ala244 side chain is in close enough proximity (4.0 Å) to the aliphatic component of the acetyl moiety of *N*-acetylserine, that its substitution by valine would sterically hinder inducer binding. The side chain of Ala247, which is further away (7.5 Å), projects in the direction of bound *N*-acetylserine such that substitution by glutamic acid is likely to hinder ligand binding. By inhibiting inducer binding, these substitutions would block CysB activation and prevent induction of the *cys* regulon. Met160 and Thr196 are part of the domain cores of EBDI and EBDII respectively and more remote from the *N*-acetylserine binding site, so that the effects of the M160I and T196I substitutions on *N*-acetylserine binding and/or *N*-acetylserine -induced conformational change are likely to be indirect.

Finally, a set of C-terminally truncated CysB variants with deletions of 19–30 residues which showed loss of repression activity, were characterised as having defects in DNA binding and oligomerisation (Lochowska et al. 2001). The crystal structures show no interchain interactions involving the C-terminal residues. Regarding DNA binding, it is possible that the C-terminal regions of the open chains, B and D, form contacts with the DNA on its passage between the BC and AD DNA binding domain pairs.

### Structural Changes Accompanying NAS Binding

To explore the structural basis of *N*-acetylserine induction of CysB, we compared the structures of CysB(88–324) bound to sulphate (CysB(88–324)-SO_4_^2−^) (Tyrrell et al. 1997) with the full length protein bound to *N*-acetylserine (CysB-NAS). In this comparison, we assume that the sulphate-bound effector-binding domain represents an uninduced state, since sulphate is not an effector of CysB. Indeed, CysB(88–324)-SO_4_^2−^ may mimic the anti-inducer state since like sulphate, the anti-inducer thiosulphate is tetrahedral and dianionic, differing only in the replacement of an oxygen by a sulphur.

Superposition of the CysB(88–324)-SO_4_^2−^protomer onto the corresponding residues from the various chains of full length CysB-NAS gives rmsΔ values of 1.4–1.5 Å for 220–230 equivalent atoms using SSM/Gesamt routines (Fig. 4B). This is significantly higher than the rmsΔ values for pairwise comparisons of subunits within and between CysB-NAS crystal forms which give rmsΔ values of 0.7 Å. The sulphate and *N*-acetylserine ligands in the respective structures occupy the pocket between the effector binding subdomains with the sulphur atom of the former and the C_α_ atom of the latter displaced by 2.8 Å (Fig. 4B). Besides polar interactions with Thr100, Thr102, Gln103 and Thr149 which are also formed by *N*-acetylserine, the sulphate ligand forms additional charge-dipole interactions with the main chain > NH group of Thr149, the side chain -OH of Thr202 and two ordered water molecules which bridge to further protein atoms (Tyrrell et al. 1997) (Fig. 4C). Following superposition of the two structures, the sulphate ligand clashes with Thr100 and Thr149 in the CysB-NAS structure, while *N*-acetylserine clashes with Thr102 and Thr202 in the CysB(88–324)-SO_4_^2−^ structure.

A dramatic alteration in structure takes place at Phe199 whose side chain projects towards the ligand in the CysB-NAS complex but away from it in CysB(88–324)-SO_4_^2−^ (Fig. 4D). The CZ atom of the Phe is displaced by 12.5 Å. In the opposite direction, the -OH of Thr202, which projects outwards from the protein surface in CysB-NAS, moves by 14 Å into the binding pocket to within hydrogen bonding distance of the sulphate (Fig. 4D). These structural changes, which take place in all four chains of the tetramer, are part of a concerted movement of residues 199–206 in the βF-αV loop in EBDII (Fig. 4B). There are further large differences in the structures at residues 150–156 in the βC-βD loop of EBDI with the C_α_ of His153 moving by 8 Å. More modest changes take place in the neighbouring βB-αIII loop and αIII helix of EBDI (Fig. 4B). These regions of the structure together form a contiguous surface on the face of the protein that forms the AC interface (Fig. 3C).

**Fig. 6:**
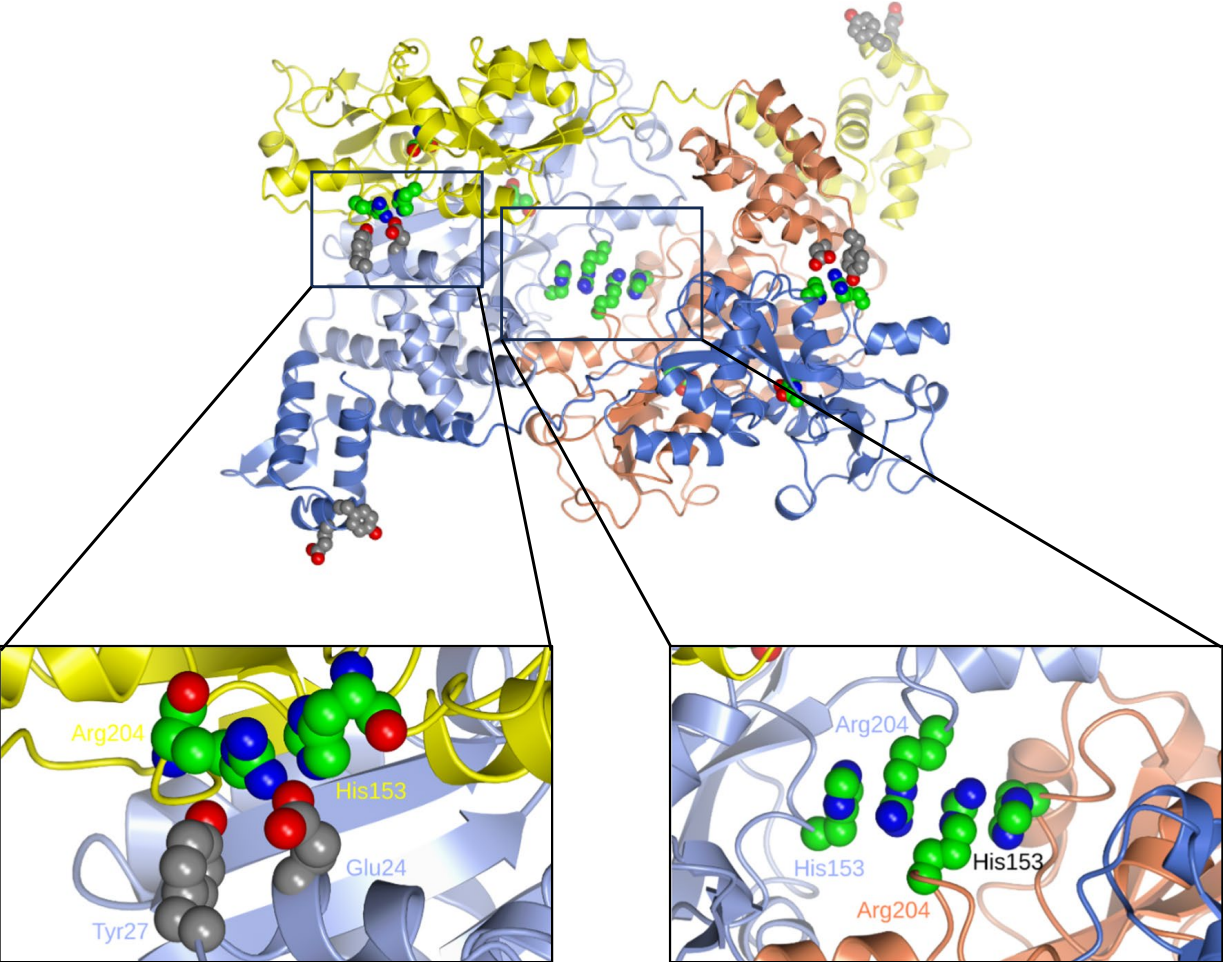
The role of Arg204. The CysB tetramer in ribbon representation with chains coloured as in Fig. 1C. The side chains of the conformationally-sensitive Arg204 and His153 residues are shown as spheres with green carbon atoms. The side chains of Glu24 and Tyr27 are shown as spheres with grey carbon atoms. The insets are enlargements of the two different His153-Arg204 environments

A striking feature of this interface in CysB-NAS is the stacking, at the heart of the tetramer, of the Arg204 and His153 side chains from each of the A and C subunits (Fig. 6). This pair of side chains within each subunit is brought into proximity by the large localised conformational changes in the βC-βD and βF-αV loops referred to above. At the pH of crystallisation (pH 8.5), the guanidinium groups of the arginines will be positively charged while the imidazole groups of the histidines are expected to be neutral. Arg-Arg pairing, as well as Arg-His interactions of this type, have been seen elsewhere and shown by molecular dynamics calculations to be stabilising (Vondrásek et al. 2009; Heyda et al. 2010). We conclude that *N*-acetylserine binding drives conformational changes in the βC-βD and βF-αV loops stabilising the inner conformation of the former and the outer conformation of the latter. In the B and D chains, where the environment of these loops is different, the guanidinium of the conformationally-sensitive Arg204 residue forms a salt-bridge to Glu24 of the DNA binding domain of chains A and C respectively, providing a possible route for propagating the effects of *N*-acetylserine binding to shape the arrangement of the DNA binding domains (Fig. 6).

As well as alteration of the AC interface described above, there are changes at the AB interface between the sulphate and NAS bound EBDs. This is evidenced by the rmsΔ value of 2.6 Å for 412 matched atoms following superposition of the EBDs from the AB dimers of the H3 crystal structure of CysB-NAS onto the crystallographic CysB(88–324)-SO4_2_^−^ dimer. This results from a relative translation, or sliding, of the interface between the A and B (or C and D) molecules by up to 6 Å leading to a rearrangement of the hydrogen bonding pattern. This indicates that the packing in these dimers is flexible which may facilitate the transitions between closed to open forms of the EBDs, which must accompany ligand binding and release.

### Comparison with the NAS-bound EBD in StCysB

The structure of an *N*-acetylserine-bound effector domain of CysB from *S. typhimurium* has been presented previously (Mittal et al. 2017). This structure differs from the structure presented here. Firstly, the Cα atom of the NAS ligand in the StCysB EBD structure is displaced by 1.3 Å from its position in the full length KaCysB structure and the orientation of the *N*-acetyl and the α-carboxylate moieties is different. Secondly, the overall structure of the StCysB EBD more closely resembles that of CysB(88–324)-SO4^2−^ than it does the structure of a CysB-NAS protomer – rmsΔ values of 1.1 Å for 235 equivalent atoms compared to 1.4 Å for 226 equivalent atoms respectively. In particular, the movement of Phe199 into the binding site and the displacement of Thr202 from it, is not observed. As a result, the *N*-acetylserine induced conformational changes of the 199–206 and 150–156 loops are not observed. This suggests that in the absence of the LH-DBD elements and the tetrameric assembly, the *N*-acetylserine bound StCysB EBD structure remains in an uninduced state. Finally, a second NAS molecule seen in the StCysB EBD structure situated close to the protein C-terminus is not present in KaCysB-NAS (Mittal et al. 2017).

### Implications for DNA Binding

**Fig. 7:**
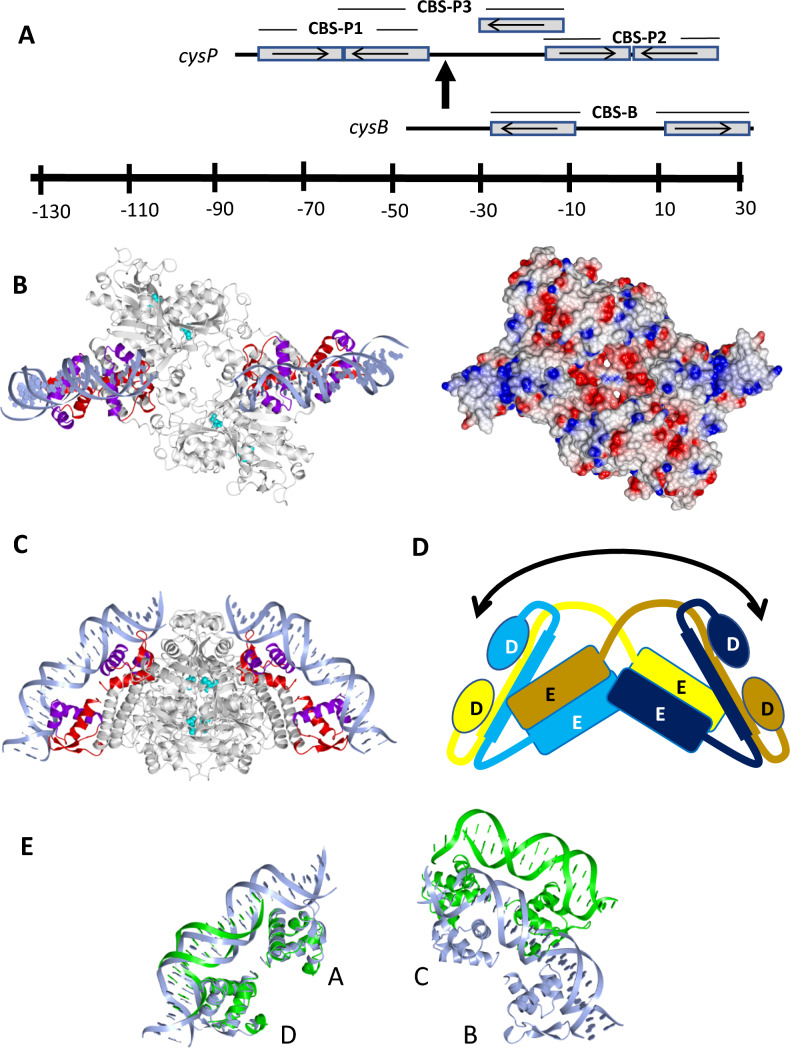
DNA Binding by CysB. **A** Schematic diagram, adapted from Colyer and Kredich (1996) of CysB binding sites (CBS) at the *cysP* and *cysB* promoters. Each box represents a 19 base pair segment earlier proposed to represent a binding site for a single CysB subunit. CBS-P1 is a transcriptional activation site where *N*-acetylserine binding to CysB stimulates DNA binding and activation of transcription, while CBS-P2 is an accessory site to which *N*-acetylserine also stimulates binding. The arrow is a site of CysB-induced bending which involves additional binding to CBS-P3. CBS-B at the *cysB* promoter is a site at which *N*-acetylserine inhibits CysB binding relieving transcriptional repression. **B** (left) and** C** Orthogonal views of a model of the full length CysB tetramer bound to DNA. To generate the model, residues 1–66 of chains AD and BC were separately superposed with the corresponding residues of the AB dimer from the BenM-DBD: DNA complex (PDB ID 4IHT). The DNA from the latter coordinate set is displayed. The wHTH elements from each chain are coloured red with the HTH in blue. In **B**, the right panel is an electrostatic surface rendering of the tetramer. The positive potential in the regions contacting the DNA backbone are evident. **D** Schematic drawing of the CysB tetramer oriented similarly to the image in **C**, coloured by subunit with DBD (D) and EBD (E) domains labelled. The curved double-headed arrow indicates the direction of the lowest frequency motion from the normal mode analysis. **E** Comparison of DNA binding in CysB and CbnR. Arrangement of the wHTH DNA binding domains in CbnR (green) and CysB (blue) with crystallographic and modelled DNA respectively. Residues 1–66 of chains A and D of CysB were superposed onto the corresponding residues of chains A and B of CbnR. The Figure shows the different position of the BC domain pair in CysB from that of the corresponding (PQ) domain pair in CbnR

The binding of CysB to DNA has been studied in vitro by electrophoretic mobility shift assays and DNase I and hydroxyl radical foot-printing methods. At the positively regulated *cysJIH*, *cysK* and *cysP* promoters, *N*-acetylserine stimulates CysB binding to a CysB-binding site (CBS) centred 50–60 bps upstream of the transcription start site and activates transcription. Each of these activation CBSs was proposed to consist of a pair of convergently oriented 19 bp half-sites exemplified by CBS-P1 in Fig. 7A. The mechanism of transcription activation is complicated however by the presence of accessory sites such as CBS-P2 and CBS-P3, and sites of *N*-acetylserine modulated DNA bending (Fig. 7A) (Hryniewicz and Kredich 1995). Meanwhile, at the *cysB* promoter, CysB was proposed to bind to two divergently oriented CBS-B half-sites which span the transcription start site and inhibit transcription (Fig. 7A). In this instance, *N*-acetylserine lowers the affinity of CysB for CBS-B thus relieving repression.

As the consensus among the CysB binding site sequences is very weak, CBSs were not assigned with high confidence. The dimensions and the arrangement of the DNA binding domain pairs in the crystal structure of the CysB tetramer are not easy to reconcile with a 19 base pair half-site which would span ~ 70 Å of B-form DNA. The distances of separation of the β-wings in the paired (AD or BC chains) wHTH domains in CysB are ~ 55 Å so that it would seem more likely that the identified 19 bp elements comprise two half-sites. The dimensions of the AD DNA binding domain pair in the CysB tetramer are similar to those of the BenM DBD dimer, which is seen in Fig. 2C bound to a 25 bp duplex spanning a core ATAC-N_7_-GTAT sequence. The DBDs of CysB and BenM have similar sequences (Fig. 2C) so it seems most likely that the two proteins would exhibit a similar mode of DNA binding. The crystal structure then suggests that the CysB tetramer would bind to two such shorter half-site pairs (Fig. 7B) separated by an intervening sequence. The length of this sequence will vary according to the extent of bending of the duplex, and the separation the AD and BC DNA binding domain pairs which in turn will be determined by the presence or absence of inducer. In the model shown in Fig. 7B, the 3’ and 5’ ends of the duplexes are separated by 35 Å indicating that they could be linked by as few as 10 additional base pairs. Thus, a duplex of 60 bp could bind to the DBDs of all four chains.

### Large-scale Motion in CysB

To investigate potential larger scale structural changes, we performed a normal mode analysis of the *N*-acetylserine bound ABCD CysB tetramer in the *H*3 crystal form using the tools available at WEBnm@. This server calculates and analyses the low frequency motions which often correlate well with functionally relevant motions in proteins (Tiwari et al. 2014). In the lowest frequency mode, the origin of motion appears to be at the twofold axis running through the core of the tetramer resulting in modest movements of the EBDs of chains A and C and larger movements of the EBDs of chains B and D. These movements radiate out to the DNA binding domains with the largest movements occurring at DBD_B_ and DBD_D_ which are furthest from the two-fold axis. Significantly, the DBD-LH_A_:DBD-LH_D_ pair undergoes concerted motions as a rigid body as does the DND-LH_B_:DBD-LH_C_ domain pair. The effect is to alter the juxtaposition of the two pairs of DNA binding domains as shown schematically in Fig. 7D.

### Comparison with CbnR

Amongst the LTTR family members, structural studies are arguably the most advanced for CbnR. While its mode of cofactor binding is not known, CbnR was the first full length LTTR structure to be determined, and at the time of writing, the only full length structure to be determined in complex with DNA (Muraoka et al. 2003b; Giannopoulou et al. 2021). In the complex with a 55 bp fragment from the *cbnR* promoter, the DNA is seen to bind to all four DNA binding domains, which have the characteristic pairwise arrangement seen across the LTTR family. The DNA in the intervening segment that connects the two binding sites is disordered. The juxtaposition of the DBD domain pairs in CbnR and CysB is different (Fig. 7D) such that the extent of curvature of the duplex required for interaction with the binding site pairs in CysB is expected to be greater than that seen in CbnR. This may reflect the absence of inducer in the CbnR-DNA complex and its presence in the CysB tetramer.

A further interesting comparative aspect of the crystal structure of CbnR is an arginine-containing loop analogous to the 199–206 loop in CysB. As seen for Arg204 in CysB, the guanidinium moieties of the side chains of Arg199 from chains A and P in CbnR pack closely together (Muraoka et al. 2003b). The functional importance of this interaction in CbnR is indicated by the constitutive (inducer-independent) expression from the *cbnA* promoter that follows mutation of this Arg199 to Ala (Moriuchi et al. 2017). This observation, and the shared presence of the Arg-Arg interaction on a loop that is conformationally sensitive in CysB, argue in favour of a commonality in the mechanism of activation upon inducer binding in these two systems.

## Concluding Remarks

The structure of full length CysB provides a framework for interpreting the rich array of genetic and biochemical studies of the regulation of cysteine biosynthesis in bacteria. It provides further insight into *N*-acetylserine binding and, through comparison with the sulphate bound EBD structure, suggests how inducer-binding is coupled to DNA binding. What is missing are structures of full length CysB in the uninduced state and a ternary complex of full length CysB bound to *N*-acetylserine and DNA.

As a LysR-type transcriptional regulator, the structure of full length CysB bound to its inducer adds to knowledge of this large and complex family. Proteins of the LTTR family control diverse processes in a wide spectrum of bacterial species. Family members originally assigned on the basis of sequence similarities (Henikoff et al. 1988) are now seen to have common tertiary structures which take up open and closed conformations during quaternary assembly (Baugh et al. 2023). Across the family, there is variety in oligomeric state and in the details of subunit interactions. Moreover, the interactions of LTTRs with DNA are complex, sometimes involving multiple promoters each with more than one operator site. Thus, it is unclear to what extent knowledge of one system can be transferred to another system. Despite the volume of work and the many advances, there is no system for which the understanding of the structural basis of LTTR action can be said to be complete. This situation may soon be remedied by electron cryomicroscopy with its more modest sample requirements and suitability for the study of large assembles that might include, the LTTR, DNA and RNA polymerase.

### Supplementary Information

Below is the link to the electronic supplementary material.

Supplementary file1 (PPTX 9180 KB)

## Data Availability

The coordinates and structure factors associated with the *H*32 and *H*3 crystal structures have been deposited in the Protein Data Bank with accession codes 9F14 and 9FDD respectively.

## Abbreviations

CBS: CysB binding site
NAS: *N*-Acetylserine
DBD: DNA Binding Domain
EBD: Effector Binding Domain
LTTR: LysR-type transcriptional regulator
