# Supplementary material for: The Structure of the LysR-type Transcriptional Regulator, CysB, Bound to the Inducer, N-acetylserine
Source: Eur Biophys J. 2024 Jul 8;53(5-6):311–26. doi: 10.1007/s00249-024-01716-w (PMC11329422; doi:10.1007/s00249-024-01716-w)
Supplement: Supplementary file 1 — Supplementary file1 (PPTX 9180 KB) [file 249_2024_1716_MOESM1_ESM.pptx]

## Slide 1
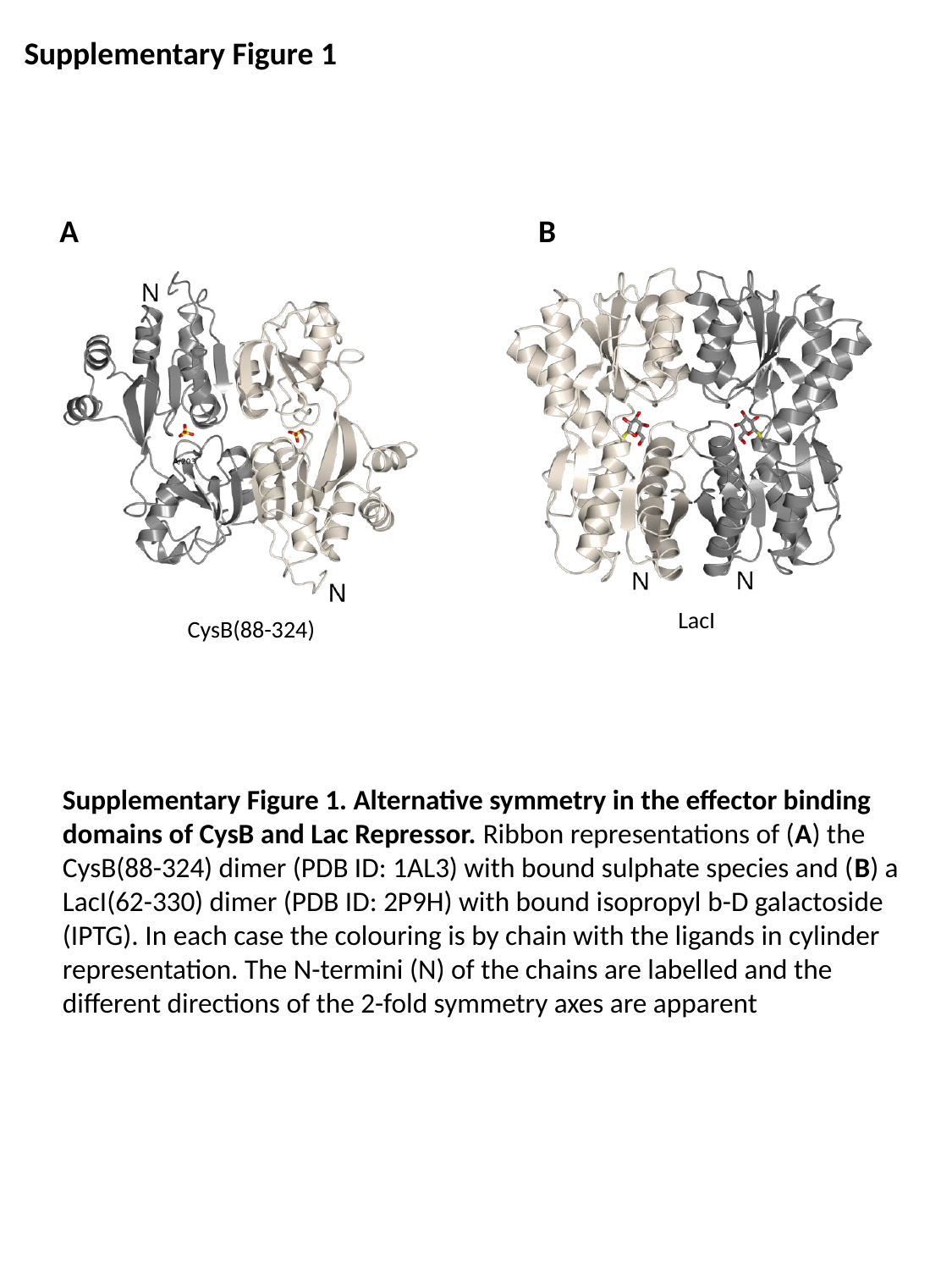

Supplementary Figure 1
B
A
LacI
CysB(88-324)
Supplementary Figure 1. Alternative symmetry in the effector binding domains of CysB and Lac Repressor. Ribbon representations of (A) the CysB(88-324) dimer (PDB ID: 1AL3) with bound sulphate species and (B) a LacI(62-330) dimer (PDB ID: 2P9H) with bound isopropyl b-D galactoside (IPTG). In each case the colouring is by chain with the ligands in cylinder representation. The N-termini (N) of the chains are labelled and the different directions of the 2-fold symmetry axes are apparent

## Slide 2
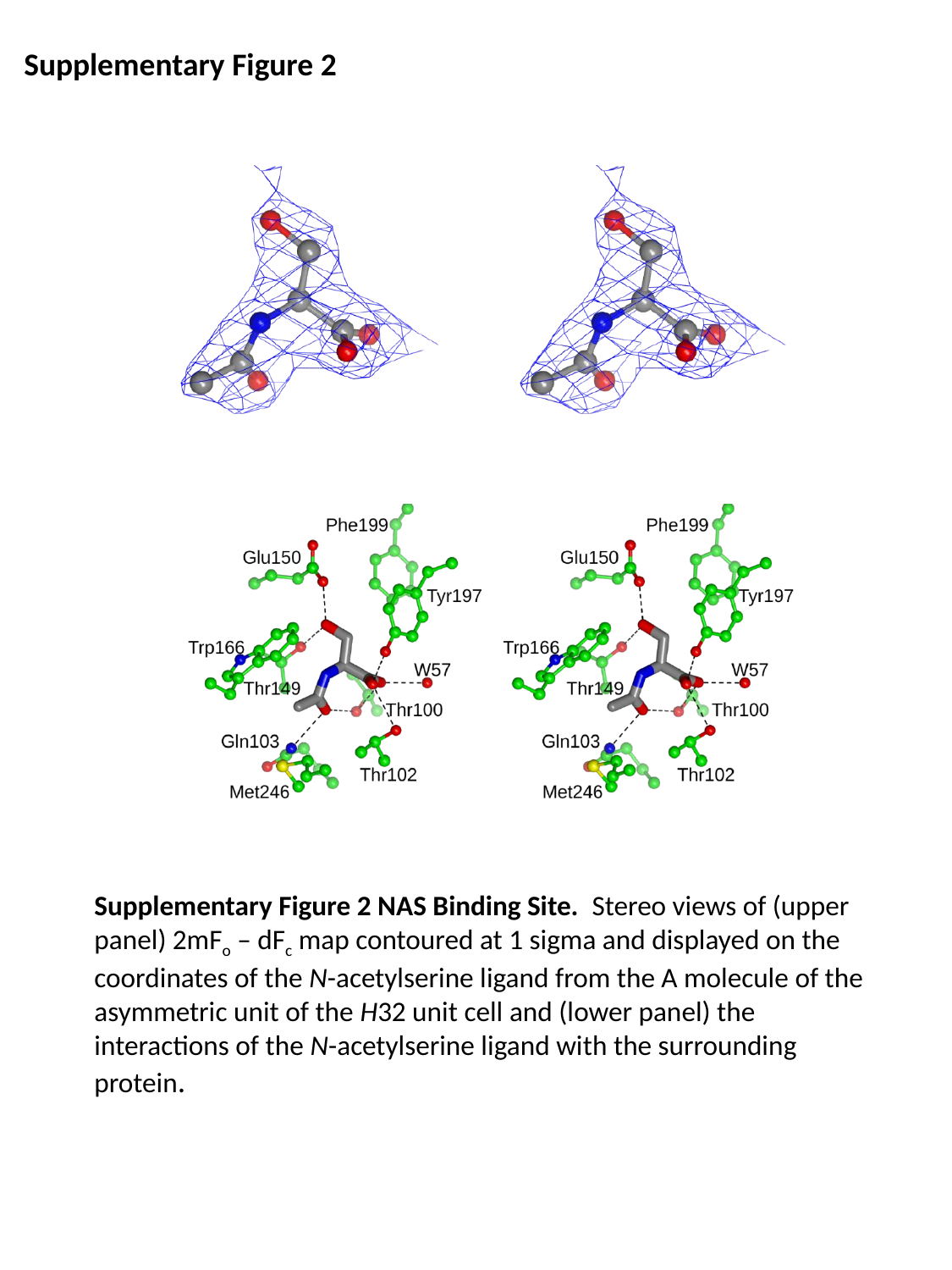

Supplementary Figure 2
Supplementary Figure 2 NAS Binding Site. Stereo views of (upper panel) 2mFo – dFc map contoured at 1 sigma and displayed on the coordinates of the N-acetylserine ligand from the A molecule of the asymmetric unit of the H32 unit cell and (lower panel) the interactions of the N-acetylserine ligand with the surrounding protein.

## Slide 3
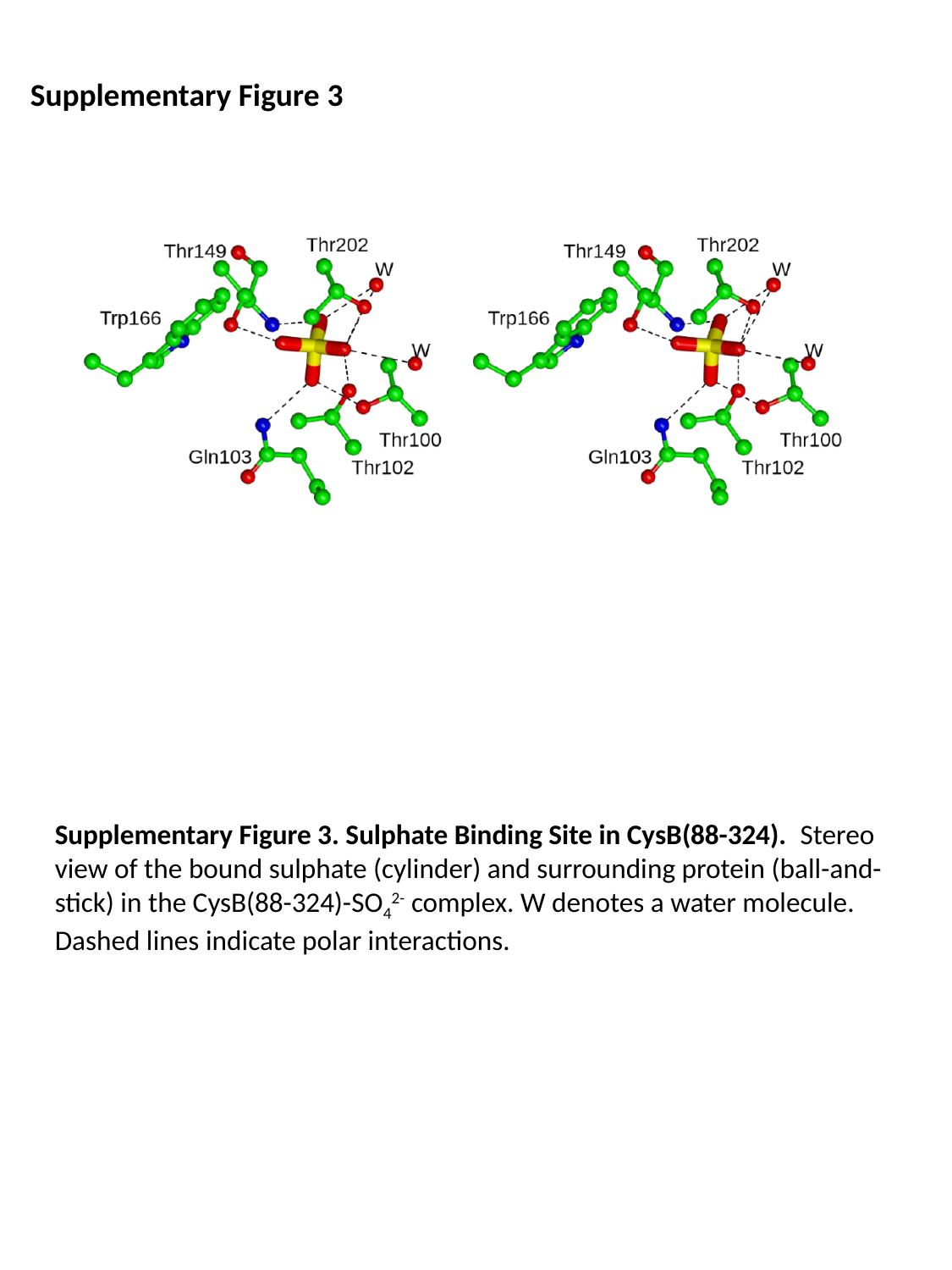

Supplementary Figure 3
Supplementary Figure 3. Sulphate Binding Site in CysB(88-324). Stereo view of the bound sulphate (cylinder) and surrounding protein (ball-and-stick) in the CysB(88-324)-SO42- complex. W denotes a water molecule. Dashed lines indicate polar interactions.
